# Multi-omics perspective: mechanisms of gastrointestinal injury repair

**DOI:** 10.1093/burnst/tkae057

**Published:** 2025-01-22

**Authors:** Haibin Zhao, Zhigang Zhang, Hongyu Liu, Mingxiu Ma, Peng Sun, Yang Zhao, Xun Liu

**Affiliations:** Department of Emergency Medicine, Shengjing Hospital of China Medical University, No. 36 Sanhao Street, Heping District, Shenyang 110004, Liaoning, China; Department of Emergency Medicine, Shengjing Hospital of China Medical University, No. 36 Sanhao Street, Heping District, Shenyang 110004, Liaoning, China; Department of Emergency Medicine, Shengjing Hospital of China Medical University, No. 36 Sanhao Street, Heping District, Shenyang 110004, Liaoning, China; Department of General Surgery, Shengjing Hospital of China Medical University, No. 36 Sanhao Street, Heping District, Shenyang 110004, Liaoning, China; Department of General Surgery, Shengjing Hospital of China Medical University, No. 36 Sanhao Street, Heping District, Shenyang 110004, Liaoning, China; Department of General Surgery, Shengjing Hospital of China Medical University, No. 36 Sanhao Street, Heping District, Shenyang 110004, Liaoning, China; Department of General Surgery, Shengjing Hospital of China Medical University, No. 36 Sanhao Street, Heping District, Shenyang 110004, Liaoning, China

**Keywords:** Gastroenterology, Multi-omics, Gastrointestinal repair, Inflammatory bowel disease

## Abstract

In this review, we examine the significance of multi-omics technologies in understanding the plethora of intricate processes that activate gastrointestinal (GI) injury repair. Multi-omics, which includes genomics, transcriptomics, proteomics, and metabolomics, allows intricate mapping of cellular responses and molecular pathways involved in GI repair. We highlight the potential of multi-omics to discover previously unknown therapeutic targets or elucidate the molecular basis of the pathogenesis of GI. Furthermore, we explore the possibilities of integrating omics data to improve prediction models, and summarize the state-of-the-art technological developments and persisting obstacles that hinder the translation of multi-omics into clinical practice. Finally, innovative multi-omics approaches that can improve patient outcomes and advance therapeutic strategies in GI medicine are discussed.

Highlights

- Multi-omics can reveal complex mechanisms of GI injury and repair, highlighting the potential for personalized medicine.
- Emerging topics like the microbiome and non-coding RNAs are key to developing new therapeutic interventions.
- Tailored multi-omics-based approaches are necessary to address the heterogeneity of GI injuries.

## Background

Gastrointestinal (GI) injuries refer to diseases that interfere with the integrity and function of the GI tract. The injuries affecting the stomach, small intestine, and colon have distinct etiologies and pathophysiological mechanisms [1,2]. Stomach injuries, including ulcers and erosions, are often caused by *Helicobacter pylori* infection, prolonged use of non-steroidal anti-inflammatory drugs (NSAIDs), or alcohol consumption, leading to mucosal damage and inflammation. Small intestine injuries, such as ulcers, erosions, and perforations, can result from infections, NSAID use, or inflammatory diseases like Crohn’s disease, with complications ranging from bleeding to sepsis. Colon injuries, commonly seen in inflammatory bowel diseases (IBDs) like ulcerative colitis (UC), involve chronic inflammation that can lead to ulcers, strictures, and an increased risk of cancer [3–5]. Overall, bacterial infections, long-term use of NSAIDs, long-term alcohol consumption, and physical impairment are the most common causes of GI injuries [5,6]. Given the high incidence of GI injuries and their effect on quality of life, it is critical to elucidate the underlying pathophysiology in these conditions in order to develop effective treatment strategies and prevent complications. Furthermore, the complexity of GI injuries due to the diversity of their anatomical sites presents a good model for studying the molecular and cellular factors involved in tissue injury and regeneration in detail at the molecular and cellular levels.

Multi-omics refers to the analyses of complex genome, transcriptome, proteome, metabolome, and epigenome data sets for a concerted study of biological processes and systems [7,8]. Genomics is the study of the entire genome and its interactions within a cell or an organism, while the transcriptomics approach studies all RNA transcripts (coding and non-coding) and their expression patterns in a spatial and temporal manner [9,10]. Proteomics and metabolomics respectively analyze the entire set of proteins (proteome) and metabolites (metabolome), especially in the context of physiological and pathological changes [11,12]. Multiomics has revolutionized the understanding of the biological pathways and networks involved in GI injuries. For instance, genomics and transcriptomics have enabled the identification of genetic variants and gene expression changes associated with the susceptibility to IBDs and treatment response [13–15]. In addition, proteomics and metabolomics have helped identify novel biomarkers for GI cancers and other GI diseases, such as irritable bowel syndrome [16–18].

The aim of this review is to explore the mechanism of GI repair from a multi-omics perspective, i.e. identifying the key genetic, transcriptomic, proteomic, and metabolomic changes that occur during the repair process, and harnessing these changes to enhance healing. We have also explored the potential of multi-omics in establishing predictive models of GI disease development and treatment response for the eventual development of personalized treatment strategies. Finally, the most recent advances, current gaps, and future research directions pertaining to multi-omics-based diagnosis, treatment, and prediction of GI injuries are discussed.

**Cellular and molecular basis of GI integrity and injury**

**Figure 1:**
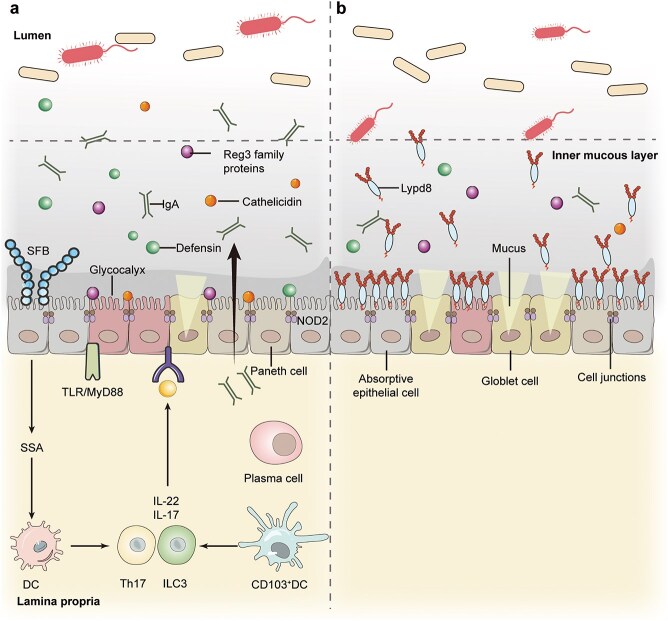
Mucosal barriers in the gut. (**a**) In the small intestine, chemical barriers like antimicrobial peptides and regenerating islet-derived 3 (Reg3) family proteins secreted by Paneth cells separate the resident microflora and intestinal epithelial cells. (**b**) In the large intestine, MUC2 and Ly6/PLAUR domain containing 8, highly glycosylated proteins secreted by goblet cells and the absorptive epithelial cells, respectively, constitute the major chemical barrier. Adapted from [55] with permission. SFB, segmentous filamentous bacteria. SAA, serum amyloid A. ILC3, type3 innate lymphoid cells. NOD2, nucleotide-binding oligomerization domain-containing 2. TLR/MyD88, toll-like receptors/myeloid differentiation factor-88

GI injury repair is driven by cellular interactions and signaling pathways involved in tissue regeneration. Epithelial cells, which form the mucosal lining of the GI tract and protect against environmental stresses and microorganisms, are the key players in post-injury tissue repair [19,20]. In addition to acting as a protective barrier, the mucosal epithelium also plays a vital role in nutrient absorption and waste secretion. This dual functionality is essential for mediating interactions between the immune system, nutrients, and microbes, and adapting to different stimuli. Studies show that any injury or dysfunction in the mucosal barrier can trigger a pathological state [21]. Tight junctions are multifunctional protein complexes that occupy the space between epithelial cells and prevent ‘leakage’ of solutes and water. Disordered tight junctions in the GI tract can promote an inflammatory milieu, potentially leading to intestinal as well as systemic diseases [22]. The mucus layer covering the GI tract is mainly comprised of the mucin proteins, and the major mucins in the intestine and stomach are MUC2 and MUC5AC, respectively [23]. Mucins are primarily secreted by the epithelial goblet cells, which play a crucial role in controlling bacterial access to epithelium and facilitating antigen presentation to the immune system. Transmembrane mucins and signaling molecules like interleukin 10 (IL-10) further regulate these interactions, highlighting a complex and not yet fully understood communication network between the epithelium and the immune system [24]. Furthermore, the intestinal macrophages can promote inflammation or tissue healing by adopting distinct phenotypes [25–27]. The chemical and physical barriers in the gut are shown in Figure 1.

At the molecular level, GI repair is governed by the Wnt, transforming growth factor β (TGF-β), Notch, and Hedgehog pathways. The Wnt signaling pathway, which plays a crucial role in the proliferation and differentiation of stem cells, facilitates tissue regeneration and restoration of the GI lining by replacing damaged epithelial cells [28]. However, chronic activation of the WNT/β-catenin pathway in aged mice exacerbated idiopathic pulmonary fibrosis by promoting cellular senescence, particularly in alveolar epithelial type II cells [29]. Sustained WNT/β-catenin pathway activity has been shown to trigger a profibrotic state in the lungs and impair lung repair and progenitor cell function [30–32]. The Notch signaling pathway is crucial to the maintenance of the intestinal stem cell (ISC) pool and a key determinant of progenitor cell fate. Kwak *et al*. showed that ghrelin reversed radiation-induced intestinal injury by restoring the epithelial barrier function and self-renewal of ISCs through the activation of the Notch signaling pathway [33]. The TGF-β pathway mediates wound healing and tissue regeneration by stimulating fibroblast proliferation and the synthesis of extracellular matrix (ECM) proteins. Nevertheless, excessive fibrosis can lead to scarring and impair tissue repair, possibly leading to other complications [34,35].

Stomach injuries, such as ulcers and erosions, often result from *Helicobacter pylori* infection or NSAID use, and tissue repair involves epithelial regeneration through the Wnt and TGF-β pathways. Small intestine injuries, including ulcers, erosions, and perforations due to infections or inflammation, rely on stem cell-driven regeneration involving the Notch and Hedgehog pathways. Colon injuries, including ulcers and inflammatory lesions from conditions like UC, involve complex interactions between epithelial cells, immune cells, and the gut microbiome that are mediated by the Wnt, TGF-β, and Hippo signaling pathways. Multi-omics strategies have helped identify the genetic, proteomic, and metabolic changes that facilitate healing, and provide targeted therapeutic interventions for each GI segment.

### Role of the microenvironment in GI healing

**Figure 2:**
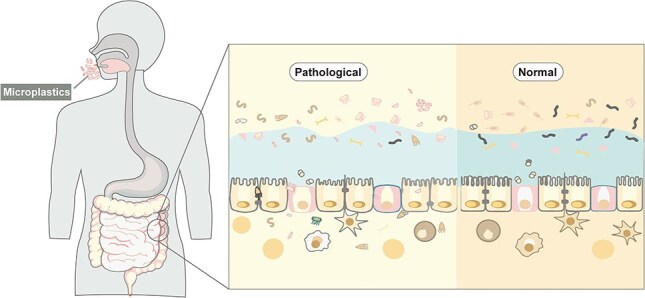
Disruption in intestinal barrier function alters the intestinal microenvironment. Adapted from [36] with permission

The GI microenvironment comprises the ECM, cytokines, and growth factors that play concerted roles during tissue healing and the regeneration process after injury (Figure 2) [36]. The ECM is a fibrous network of collagen and fibronectin that not only provides mechanical strength to the tissues and but also regulates the behavior of the resident cells. For instance, the impact of ECM on endothelial cell function and vascular development is a key factor in tumorigenesis, and offers the possibility of developing targeted therapies that may improve patient outcomes [37]. ECM remodeling during GI injury promotes tissue regeneration by promoting the migration and proliferation of various cell types. Destruction of ECM components can impair tissue healing or stimulate excessive fibrosis, potentially leading to chronic GI diseases [37–39]. High ECM1 expression in macrophages induces M1 polarization and exacerbates inflammation, and targeting ECM1 alleviated the symptoms of IBD in a mouse model [40].

Cytokines within the GI microenvironment are secreted by immune cells, epithelial cells, and other resident cells in response to injury [41], and coordinate the inflammatory response to initiate tissue healing, and remove pathogens and debris. Tumor necrosis factor (TNF)-α and IL-6 promote inflammation and the transition to tissue repair [42]. Dietary palmitoleic acid has been shown to enhance the efficacy of anti-TNF-α therapy in IBD patients by repairing the gut mucosa, mitigating the inflammatory response, and selectively promoting beneficial host microbiota [43]. The Hedgehog signaling pathway regulates the growth, immune tolerance, and drug resistance of GI tumors, and its aberrant activation contributes to the survival and therapeutic resistance of cancer stem cells. Therefore, targeting this pathway may offer a therapeutic option by normalizing the tumor environment [44–46]. Epithelial growth factor and vascular endothelial growth factor synergistically promote tissue repair by regulating the proliferation of epithelial cells and endothelial cells [47–49]. Vascular endothelial growth factor was first identified for its role in angiogenesis, and also acts directly on GI tumor cells through the receptors expressed on the latter [49,50]. Increased angiogenesis during healing ensures the delivery of nutrients and oxygen necessary for tissue regeneration. Overall, the complex interplay between ECM components, cytokines, and growth factors within the GI environment drives the immediate response to injury and shapes the outcomes of tissue repair and regeneration.

### Genomics and transcriptomics in GI injury and repair

Genomics and transcriptomics can provide insight into the changes in gene expression that occur during GI injury and subsequent repair (Figure 1). Genomics focuses on identifying genetic variants and alterations that may predispose individuals to more severe GI damage or affect the healing process [51]. For instance, comparing the genomes of patients with different responses to similar GI injuries can unearth specific genes that contribute to resilience or susceptibility to injury [14]. A study using Illumina-based metagenomic sequencing characterized 3.3 million unique microbial genes from the fecal samples of 124 European individuals, which represents the common microbial genes in the human gut, 99% of which belong to bacterial species. These findings suggest that bacterial genes are largely shared among individuals, and each person harbors at least 160 bacterial species in their gut. Therefore, the core functions of the minimal gut metagenome and bacterial genome, which are present in all individuals and most bacteria, can be defined based on these studies [52–54]. For instance, variations in genes associated with the immune system or cell adhesion can significantly affect an individual’s response to GI inflammatory triggers or bodily injuries [55,56]. In addition, specific metabolite changes are linked to disease severity and activity, such as altered bile acids in stool and increased polyamines in the plasma.

Analyzing the transcriptomes of cells or tissues at different stages of damage and repair provides critical information regarding the biological processes and pathways that are activated at specific time points following an injury, along with the molecular mechanisms of inflammation, cell proliferation, migration, and differentiation during tissue repair [57]. Intestinal metaplasias in the esophagus (BE-IM) and stomach (GIM) are precursors to cancer, and share transcriptional traits related to fibroblast activation, suggesting potential for common detection and treatment strategies [58]. In a previous study, we explored the cellular and molecular landscape of the gastric tumor microenvironment (TME) by analyzing the bulk and single-cell RNA sequencing of 24 patients from The Cancer Genome Atlas. Based on the transcriptomics data, we identified 81 distinct cell types within the TME, mainly activated fibroblasts and endothelial cells. Fibroblasts are the predominant population in tumors and contribute to an immunosuppressive environment that is related to poor prognosis and resistance to anti-programmed cell death protein 1 (PD1) therapy. In addition, we also detected Interferon-gamma (IFN γ)-activated T cells and HLA-II-expressing macrophages that were positively correlated with better treatment response and longer survival. These findings provide new insights into the cellular mechanisms of gastric cancer, and a platform for identifying new diagnostic and therapeutic biomarkers [59]. We assessed the microenvironment of gastric signet-ring cell carcinoma using targeted single-cell RNA sequencing and protein expression profiling, and found poor infiltration of CD4+ and CD8+ T cells, and low abundance of B cells [60]. In addition, we also identified CXCL13, a chemokine produced by distinct subsets of T cells, as the determinant of immune quiescence in the TME. Therefore, CXCL13 can serve as a biomarker for selecting gastric signet-ring cell carcinoma patients most likely to benefit from immune checkpoint therapy [61,62].

**Figure 3:**
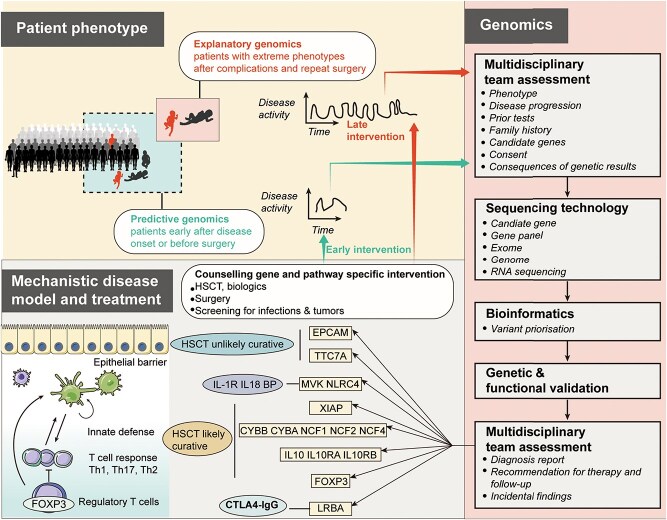
Clinical genomics in the management of Mendelian disorder-associated IBD. Adapted from [14] with permission. HSCT, hematopoietic stem cell transplantation. XIAP, X-linked inhibitor of apoptosis protein. EPCAM, Epithelial cell adhesion molecule. TTC7A, tetratricopeptide repeat domain 7A. MVK, mevalonate kinase. NLRC4, NLR-family CARD-containing protein 4. CYBB, cytochrome B-245 beta chain. CYBA, cytochrome B-245 alpha chain. NCF2, neutrophil cytosolic factor 2. NCF4, neutrophil cytosolic factor 4. FOXP3, forkhead box p3. LRBA, LPS responsive beige-like anchor protein. CTLA4, cytotoxic T-lymphocyte associated protein 4

Clinical genomics has also been applied to detect causal genetic variants of Mendelian disorder-associated IBD to facilitate early detection, prevent severe complications, and establish genetic tests for family counseling (Figure 3).

### Proteomics and metabolomics: decoding protein expression and metabolic shifts in GI repair

Proteomics and metabolomics can provide significant insights into protein expression patterns and metabolic changes that occur during GI damage and repair (Figure 2). Proteome-wide Mendelian randomization and colocalization analyses have identified macrophage stimulating 1 (MST1) and (hepatocyte growth factor activator (HGFAC) as novel risk factors for IBD and UC, and warrant further investigation as potential therapeutic targets [63]. Furthermore, integrated multi-omics analyses have shown that the mitochondrial proteins PARK7 and ACADM are associated with reduced risks of IBD and UC, and the gene methylation and expression levels and disease risks are inversely correlated [64]. Several potential therapeutic targets of IBD and its subtypes have been identified in recent years, including inflammatory cytokines and chemokines, pro-regenerative growth factors, and enzymes that degrade the ECM. The chronic colon inflammation in IBD is the result of Heat shock factor 1 (HSF1)-dependent ECM remodeling and may progress to colorectal cancer (CRC). The proteomic changes accompanying the reorganization of the ECM have been identified in both murine models and CRC patients [65]. Lin *et al*. showed that milk fat globule-epidermal growth factor (8MFGE8) can reverse intestinal fibrosis in Crohn’s disease and prevent intestinal strictures in IBD by inhibiting ECM production via the integrin-mediated signaling pathway [66]. Thus, monitoring the temporal dynamics of the proteome during healing can identify potential biomarkers of pathological changes and tissue repair.

**Figure 4:**
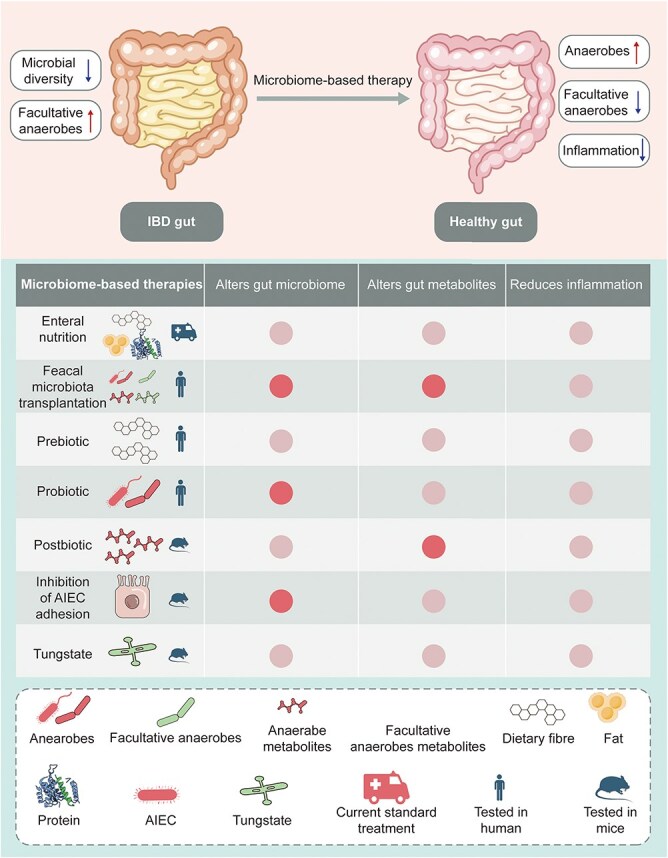
Microbiome-based therapies for IBD. Adapted from [7] with permission. AIEC, adhesive invasive *E. coli*

The energy and biosynthetic requirements for tissue regeneration are met through metabolic adaptations. Untargeted metabolomics and shotgun metagenomics analyses of the intestinal samples from IBD patients have revealed significant changes in the sphingolipids, bile acids, and triglycerides, which correlated with specific microbial species. The altered metabolite profile may contribute to gut inflammation and oxidative stress adaptation in IBD, and the robust association between the gut microflora and metabolites offers potential diagnostic and therapeutic targets for IBD management [67]. Halfvarson *et al*. showed that the gut microbiota of IBD patients had greater dynamic variability compared to that of healthy individuals, particularly in the patients with ileal Crohn’s disease following surgical resection. While inflammation did not correlate directly with microbial dysbiosis, the fluctuations in the composition of the microbiota were associated with increased use of therapeutic drugs during disease flares. Thus, stabilizing the gut microbiome is a viable strategy to maintain remission in IBD [68]. Glycolytic metabolites tend to increase during the initial inflammatory phase of IBD, reflecting a surge in energy requirements. As tissue repair progresses, cell proliferation and matrix reconstruction are predominantly supported by altered lipid metabolism and amino acid biosynthesis. Butyrate mitigates the functions of neutrophils in IBD by downregulating the synthesis of pro-inflammatory mediators and inhibiting the formation of neutrophil extracellular traps, indicating its therapeutic potential in alleviating mucosal inflammation through immunomodulatory effects [69]. In the PROTECT study, the gut microbiomes of 405 pediatric UC patients were analyzed to assess the role of the microbiota in disease progression and treatment response. The researchers observed a lack of core gut microbes and an increase in bacteria typically found in the oral cavity, which correlated with more severe disease in the initial stages. In addition, changes in the abundance of specific microbes over time were predictive of remission or persistent disease after treatment. Increased microbial diversity was also observed before surgical removal of the colon. These findings suggest that the microbiome interacts with host factors in UC, and elucidating the underlying mechanisms could lead to better treatment strategies [70,71]. A study of 19 pediatric patients with newly diagnosed IBD and 10 healthy controls showed increased inflammation and gut dysbiosis in patients with Crohn’s disease and UC compared to healthy individuals, and the changes were more pronounced in the UC patients. Although the overall microbial imbalance correlated with disease severity, it did not predict treatment response. Nevertheless, differences in specific bacterial genera were identified between the responders and non-responders, and the pre-treatment microbiome profiles could predict outcomes with about 76.5% accuracy. This suggests that the composition of the gut microbiome is a predictive factor of the therapeutic response of IBD patients [72–74]. Microbiome-based therapies for IBD are designed to restore the gut microbiota by increasing microbial diversity, and increasing the abundance of anaerobic bacteria while reducing that of facultative anaerobes to diminish gut inflammation (Fig. 4). Therapies targeting the microbiome include dietary changes, probiotic supplementation, and direct targeting of pathogenic bacteria and their metabolic pathways. The impact of these therapies on IBD can be both direct and indirect, influencing gut inflammation, microbial communities, and metabolic profiles.

Taken together, proteomics and metabolomics not only map the biochemical landscape of GI repair, but also highlight the interactions between metabolic pathways and protein networks. This integrated view enables a more targeted approach to the treatment of GI diseases, which is aimed at enhancing natural repair mechanisms or alleviating complications.

### Epitranscriptomics in GI repair and regeneration

During tissue regeneration, RNA modifications facilitate the dynamic changes in gene expression required for cell growth and differentiation. Epitranscriptomics is the study of global epigenetic changes in RNA transcripts that affect gene expression profiles. Post-transcriptional RNA modifications, such as N6-methyladenosine (m6A), play a vital role in various biological processes, including tissue damage repair, stem cell proliferation and differentiation, and tissue regeneration. By fine-tuning the expression of genes involved in cell cycle progression, apoptosis, and extracellular matrix remodeling, these epigenetic modifications play a critical role in orchestrating the complex process of tissue regeneration. Advanced sequencing technologies such as ASm6A analysis and m6A single-base sequencing have shown that these epigenetic modifications influence mRNA stability, translation, and splicing, which in turn are crucial for the timely production of proteins involved in tissue repair. For example, m6A modification can enhance the stability and translation of mRNAs encoding proteins that mediate inflammatory responses and wound healing, thereby accelerating the repair process. Furthermore, global RNA methylation has also been associated with the proliferation and differentiation of ISCs, a key factor in the regeneration of the GI epithelium following injury.

Integrating epitranscriptomics with multi-omics data can provide a more comprehensive understanding of how RNA modifications interact with genomic, transcriptomic, proteomic, and metabolomic changes to coordinate the response to tissue injury, and may even lead to the identification of novel biomarkers and therapeutic targets. For example, aberrant RNA modifications may serve as biomarkers for disease progression or treatment response in IBD or CRC. In addition, RNA-modifying enzymes, such as methyltransferases and demethylases, can be promising therapeutic targets to enhance tissue repair and regeneration. Thus, it is possible to enhance the regenerative capacity of tissues or mitigate pathological processes that hinder repair by influencing the epitranscriptomic landscape.

### Challenges and systems biology approaches in GI research

The integration of multi-omics data can converge diverse data streams to accurately predict complex biological behaviors or treatment responses. The goal of such models is to develop a well-defined network of molecular interactions involved in a biological process, such as tissue repair in the GI system. For example, a protein–protein interaction network related to the wound healing process may be useful in identifying the hub genes or metabolic pathways at different time points, which often represent potential targets for therapeutic intervention. However, integrating such diverse and massive data sets poses huge challenges, such as heterogeneity of data types, variations in the scale of measurements, and complexity of biological interdependencies.

### Multi-omics in GI repair mechanisms

Multi-omics approaches have significantly improved our understanding of the mechanisms of repair and regeneration in the GI tract, and identified potential therapeutic targets. Some of the key research findings of integrating multiple omics data sets are discussed in this section.

One landmark study used genomics and transcriptomics to explore the response of the GI epithelium to inflammatory damage in Crohn’s disease patients. Salas *et al*. identified a unique gene-expression profile that was linked to successful mucosal healing as opposed to persistent inflammation, and identified possible genetic markers of treatment response. In addition, the study also elucidated the role of certain signal transduction pathways, like the JAK–STAT pathway, in tissue repair [75]. Several studies have used proteomics and metabolomics to assess metabolic changes during gastric ulcer repair [76,77]. For instance, one study showed that the expression levels of proteins related to energy metabolism and cell proliferation surged at the ulcer site, and was accompanied by changes in lipid and amino acid metabolism. The findings further suggest that healing might be quickened through targeted metabolic interventions that provide the essential energy matrix and building blocks for cellular and tissue reconstitution. Furthermore, another study integrated genomics, transcriptomics, and proteomics data to show that changes in the gut microbiota, gene expression, and protein synthesis affect the gut inflammatory response and healing, thereby underscoring the potential of microbiome modulation as a therapeutic strategy for GI injuries [67,78,79].

Recent advances in multiomics have facilitated the discovery of new biological pathways and therapeutic targets for GI repair, heralding a new era of potential interventions. Furthermore, omics approaches have elucidated the complex interactions between the host microbiome and the immune system that influence inflammation and treatment outcomes. For instance, several microbe-derived metabolites have been identified that regulate the proliferation and differentiation of host epithelial cells, offering new therapeutic targets to enhance mucosal healing through microbiome manipulation [80,81]. Furthermore, Chen *et al*. identified the endoplasmic reticulum stress kinase protein kinase RNA-like endoplasmic reticulum kinase (PERK) as a receptor for the gut-microbe-derived metabolite trimethylamine N-Oxide (TMAO), and showed that PERK activation drives the transcription factor forkhead box transcription factor O1 (FoxO1). Reduction in TMAO levels via enzymatic inhibition or modulation of gut microbiota inhibited PERK activation and FoxO1 expression, indicating that TMAO is involved in the pathogenesis of metabolic syndrome [82]. Transcriptomics studies have also identified several non-coding RNAs, including microRNAs and long-chain non-coding RNAs, that regulate the expression of genes involved in GI injury and repair. Navarro-Corcuera *et al*. showed that long-chain non-coding RNA ACTA2-AS1 facilitates the recruitment of the epigenetic co-activator p300 and transcription factor ETS Transcription Factor ELK1 (ELK1) to the promoters of pro-fibrogenic genes, resulting in enhanced histone acetylation and transcription of these genes in biliary diseases. Cholangiocyte-selective knockout or inhibition of p300 blocked the action of ACTA2-AS1, thus reducing ductular reaction and fibrosis [83]. There is evidence that TGF-β promotes fibrogenic gene transcription in cholangiocytes by inducing proteasomal degradation of the epigenetic regulator enhancer of zeste homolog 2 (EZH2) through K63-linked ubiquitination. The resulting activation of hepatic stellate cells enhanced biliary fibrosis, indicating that proteasome inhibition could mitigate these effects [84,85]. These studies offer new avenues for targeted therapies that modulate non-coding RNAs to improve gut healing.

Proteomics-based studies in recent years have identified previously unrecognized signaling pathways in GI repair. For example, proteomic and genomic data indicate that the Hippo signaling pathway regulates organ size by controlling cell proliferation and apoptosis [86–88]. In addition, the Hippo pathway has also been implicated in mucosal regeneration and barrier function, indicating its potential as a target for enhancing tissue repair and preventing fibrosis [89,90]. Taken together, multi-omics analyses have advanced our understanding of GI repair mechanisms. By dissecting the complex molecular and cellular interactions that occur during healing, multi-omics offers a promising avenue for developing more personalized and effective treatment strategies.

### Translation of multi-omics findings to therapeutic strategies for GI diseases

The clinical translation of the therapeutic possibilities obtained from multi-omics studies could significantly impact the management and outcomes of GI diseases. Multi-omics studies require robust bioinformatics pipelines for data preprocessing, alignment, assembly, variant calling, and quantification, utilizing tools such as FastQC, BWA, Bowtie2, GATK, and HTSeq. Statistical frameworks like DESeq2, EdgeR, WGCNA, and machine learning models are crucial for interpreting complex data. Integration methods like MOFA and iCluster facilitate combining data from different omics layers. Proper data normalization and standardization, including batch effect correction with ComBat and sva, scaling, transformation, and cross-platform normalization, ensure comparability and reduce technical variability. Integration of genomic, transcriptomic, proteomic, and metabolomic data will be instrumental in predicting specific molecular profiles that lead to treatment response, resulting in more individualized therapies with high efficacy and lower side effects.

Biomarkers identified through multi-omics analysis have been applied in the early diagnosis and monitoring of GI damage. Proteomics and metabolomics in particular can reveal unique signatures of damage before clinical symptoms appear. In addition, multiomics has now permitted the discovery of newer drug targets based on hitherto unknown GI repair pathways. For example, transcriptomic analysis may reveal upregulation of certain pathways during GI injury that can be pharmacologically manipulated to increase tissue regeneration and lower scar formation.

### Advancements in multi-omics technologies: innovations and challenges in GI research

Emerging multi-omics technologies can significantly improve the resolution and depth of research in the field of GI diseases, although there are inherent challenges and limitations that may hinder its clinical translation. For example, single-cell sequencing allows dissection of molecular mechanisms at the cellular level within heterogeneous tissues [91–93], which can provide a detailed understanding of cell-specific responses during GI injury and repair, potentially revealing new therapeutic targets [94–97]. Single-cell multi-omics has the potential to improve diagnosis, treatment, and prevention of GI diseases.

Multi-omics has also accelerated the development of personalized medicine through the high-throughput discovery of early diagnosis and prognosis biomarkers, and therapeutic targets. Tailored treatments based on an individual’s genetic and molecular profile can minimizes adverse effects and enhance outcomes. Several proteomic and metabolomic studies have identified markers for the early detection of colorectal cancer and predicted flare-ups in UC and Crohn’s disease. Furthermore, integrating multi-omics data improves predictive models for disease progression and treatment response, while microbiome-based therapies offer new avenues for managing chronic GI conditions.

One major issue in multi-omics studies is the high dimensionality and complexity of the datasets, which can lead to difficulties in data interpretation. The sheer volume of data increases the risk of false positives and makes it challenging to distinguish causality from mere correlation. This issue underscores the importance of rigorous validation strategies to confirm findings. Potential solutions include conducting replication studies to ensure that results are reproducible across different cohorts, and using orthogonal methods that provide independent confirmation of the results. For example, findings from genomic analyses could be validated through proteomic or metabolomic approaches to establish more robust causal links.

## Conclusions

This review explores the key role of multi-omics techniques in revealing the mechanisms of GI injury and repair. By integrating genomics, transcriptomics, proteomics, and metabolomics, the study reveals the dynamic processes of GI pathology and rehabilitation, and highlights the potential impact of gut microbiota and non-coding RNAs in disease progression. Although multi-omics technology shows promise for personalized therapy, its clinical application still faces challenges in data management and translation. Future research should focus on real-time tracking of microbiome and host interactions and longitudinal analysis of the effects of chronic inflammation on disease progression, thereby driving the clinical translation of multiomics in gastroenterology and the development of personalized therapy.

## References

[1] Rubio-Tapia A, Hill ID, Semrad C, Kelly CP, Greer KB, Limketkai BN. et al. American College of Gastroenterology guidelines update: diagnosis and management of celiac disease. *Am J Gastroenterol* 2023;118:59–76. 10.14309/ajg.0000000000002075. 36602836

[2] Birk M, Bauerfeind P, Deprez PH, Häfner M, Hartmann D, Hassan C. et al. Removal of foreign bodies in the upper gastrointestinal tract in adults: European Society of Gastrointestinal Endoscopy (ESGE) clinical guideline. *Endoscopy* 2016;48:489–96. 10.1055/s-0042-100456. 26862844

[3] Moayyedi P, Axon AT. Endoscopy and gastric ulcers. *Endoscopy* 1995;27:689–93. 10.1055/s-2007-1005789. 8903984

[4] Han Y, Liao Z, Li Y, Zhao X, Ma S, Bao D. et al. Magnetically controlled capsule endoscopy for assessment of antiplatelet therapy-induced gastrointestinal injury. *J Am Coll Cardiol* 2022;79:116–28. 10.1016/j.jacc.2021.10.028. 34752902

[5] Bjarnason I, Scarpignato C, Holmgren E, Olszewski M, Rainsford KD, Lanas A Mechanisms of damage to the gastrointestinal tract from nonsteroidal anti-inflammatory drugs. *Gastroenterology* 2018;154:500–14. 10.1053/j.gastro.2017.10.049. 29221664

[6] Goldenring JR, Mills JC. Cellular plasticity, reprogramming, and regeneration: metaplasia in the stomach and beyond. *Gastroenterology* 2022;162:415–30. 10.1053/j.gastro.2021.10.036. 34728185 PMC8792220

[7] Schirmer M, Garner A, Vlamakis H, Xavier RJ Microbial genes and pathways in inflammatory bowel disease. *Nat Rev Microbiol* 2019;17:497–511. 10.1038/s41579-019-0213-6. 31249397 PMC6759048

[8] Sun C, Wang A, Zhou Y, Chen P, Wang X, Huang J. et al. Spatially resolved multi-omics highlights cell-specific metabolic remodeling and interactions in gastric cancer. *Nat Commun* 2023;14:2692. 10.1038/s41467-023-38360-5. 37164975 PMC10172194

[9] Zheng W, Zhao S, Yin Y, Zhang H, Needham DM, Evans ED. et al. High-throughput, single-microbe genomics with strain resolution, applied to a human gut microbiome. *Science* 2022;376:eabm1483. 10.1126/science.abm1483. 35653470

[10] Almeida A, Nayfach S, Boland M, Strozzi F, Beracochea M, Shi ZJ. et al. A unified catalog of 204,938 reference genomes from the human gut microbiome. *Nat Biotechnol* 2021;39:105–14. 10.1038/s41587-020-0603-3. 32690973 PMC7801254

[11] Islam , Khan MZ, Tam SY, Law HKW. Advances in high throughput proteomics profiling in establishing potential biomarkers for gastrointestinal cancer. *Cells* 2022;11:973–90. 10.3390/cells11060973. PMC8946849 35326424

[12] Huang CH, Chiou SH. Clinical proteomics identifies potential biomarkers in helicobacter pylori for gastrointestinal diseases. *World J Gastroenterol* 2014;20:1529–36. 10.3748/wjg.v20.i6.1529. 24587628 PMC3925861

[13] Plevris N, Lees CW. Disease monitoring in inflammatory bowel disease: evolving principles and possibilities. *Gastroenterology* 2022;162:1456–1475.e1. 10.1053/j.gastro.2022.01.024. 35101422

[14] Uhlig HH, Muise AM. Clinical genomics in inflammatory bowel disease. *Trends Genet* 2017;33:629–41. 10.1016/j.tig.2017.06.008. 28755896

[15] Sands BE . Biomarkers of inflammation in inflammatory bowel disease. *Gastroenterology* 2015;149:1275–1285.e2. 10.1053/j.gastro.2015.07.003. 26166315

[16] Abyadeh M, Meyfour A, Gupta V, Zabet Moghaddam M, Fitzhenry MJ, Shahbazian S. et al. Recent advances of functional proteomics in gastrointestinal cancers- a path towards the identification of candidate diagnostic, prognostic, and therapeutic molecular biomarkers. *Int J Mol Sci* 2020;21:8532–57. 10.3390/ijms21228532. PMC7697099 33198323

[17] Roshani M, Molavizadeh D, Sadeghi S, Jafari A, Dashti F, Mirazimi SMA. et al. Emerging roles of miR-145 in gastrointestinal cancers: a new paradigm. *Biomed Pharmacother* 2023;166:115264. 10.1016/j.biopha.2023.115264. 37619484

[18] Shi W, Wang Y, Xu C, Li Y, Ge S, Bai B. et al. Multilevel proteomic analyses reveal molecular diversity between diffuse-type and intestinal-type gastric cancer. *Nat Commun* 2023;14:835. 10.1038/s41467-023-35797-6. 36788224 PMC9929250

[19] Pelaseyed T, Bergström JH, Gustafsson JK, Ermund A, Birchenough GM, Schütte A. et al. The mucus and mucins of the goblet cells and enterocytes provide the first defense line of the gastrointestinal tract and interact with the immune system. *Immunol Rev* 2014;260:8–20. 10.1111/imr.12182. 24942678 PMC4281373

[20] Ogulur I, Pat Y, Aydin T, Yazici D, Rückert B, Peng Y. et al. Gut epithelial barrier damage caused by dishwasher detergents and rinse aids. *J Allergy Clin Immunol* 2023;151:469–84. 10.1016/j.jaci.2022.10.020. 36464527

[21] Turner JR . Intestinal mucosal barrier function in health and disease. *Nat Rev Immunol* 2009;9:799–809. 10.1038/nri2653. 19855405

[22] Suzuki T . Regulation of intestinal epithelial permeability by tight junctions. *Cell Mol Life Sci* 2013;70:631–59. 10.1007/s00018-012-1070-x. 22782113 PMC11113843

[23] Johansson ME, Sjövall H, Hansson GC. The gastrointestinal mucus system in health and disease. *Nat Rev Gastroenterol Hepatol* 2013;10:352–61. 10.1038/nrgastro.2013.35. 23478383 PMC3758667

[24] Dokoshi T, Chen Y, Cavagnero KJ, Rahman G, Hakim D, Brinton S. et al. Dermal injury drives a skin to gut axis that disrupts the intestinal microbiome and intestinal immune homeostasis in mice. *Nat Commun* 2024;15:3009. 10.1038/s41467-024-47072-3. 38589392 PMC11001995

[25] Pan X, Zhu Q, Pan LL, Sun J Macrophage immunometabolism in inflammatory bowel diseases: from pathogenesis to therapy. *Pharmacol Ther* 2022;238:108176. 10.1016/j.pharmthera.2022.108176. 35346728

[26] Bain CC, Mowat AM. Macrophages in intestinal homeostasis and inflammation. *Immunol Rev* 2014;260:102–17. 10.1111/imr.12192. 24942685 PMC4141699

[27] Gabanyi I, Muller PA, Feighery L, Oliveira TY, Costa-Pinto FA, Mucida D Neuro-immune interactions drive tissue programming in intestinal macrophages. *Cell* 2016;164:378–91. 10.1016/j.cell.2015.12.023. 26777404 PMC4733406

[28] Si X, Jia H, Liu N, Li J, Pan L, Wang J. et al. Alpha-Ketoglutarate attenuates colitis in mice by increasing lactobacillus abundance and regulating stem cell proliferation via Wnt-hippo Signaling. *Mol Nutr Food Res* 2022;66:e2100955. 10.1002/mnfr.202100955. 35220672

[29] Yildiz S, Kinali M, Wei JJ, Milad M, Yin P, Adli M. et al. Adenomyosis: single-cell transcriptomic analysis reveals a paracrine mesenchymal-epithelial interaction involving the WNT/SFRP pathway. *Fertil Steril* 2023;119:869–82. 10.1016/j.fertnstert.2023.01.041. 36736810 PMC11257082

[30] Lehmann M, Hu Q, Hu Y, Hafner K, Costa R, van den Berg A. et al. Chronic WNT/β-catenin signaling induces cellular senescence in lung epithelial cells. *Cell Signal* 2020;70:109588. 10.1016/j.cellsig.2020.109588. 32109549 PMC8968687

[31] Katoh M . Canonical and non-canonical WNT signaling in cancer stem cells and their niches: cellular heterogeneity, omics reprogramming, targeted therapy and tumor plasticity (review). *Int J Oncol* 2017;51:1357–69. 10.3892/ijo.2017.4129. 29048660 PMC5642388

[32] Chen X, Tan H, Xu J, Tian Y, Yuan Q, Zuo Y. et al. Klotho-derived peptide 6 ameliorates diabetic kidney disease by targeting Wnt/β-catenin signaling. *Kidney Int* 2022;102:506–20. 10.1016/j.kint.2022.04.028. 35644285

[33] Kwak SY, Shim S, Park S, Kim H, Lee SJ, Kim MJ. et al. Ghrelin reverts intestinal stem cell loss associated with radiation-induced enteropathy by activating notch signaling. *Phytomedicine* 2021;81:153424. 10.1016/j.phymed.2020.153424. 33278782

[34] Morikawa M, Derynck R, Miyazono K. TGF-β and the TGF-β family: context-dependent roles in cell and tissue physiology. *Cold Spring Harb Perspect Biol* 2016;8:a021873–a021897. 10.1101/cshperspect.a021873. PMC4852809 27141051

[35] Zhang T, Wang XF, Wang ZC, Lou D, Fang QQ, Hu YY. et al. Current potential therapeutic strategies targeting the TGF-β/Smad signaling pathway to attenuate keloid and hypertrophic scar formation. *Biomed Pharmacother* 2020;129:110287. 10.1016/j.biopha.2020.110287. 32540643

[36] Huang Z, Weng Y, Shen Q, Zhao Y, Jin Y. Microplastic: A potential threat to human and animal health by interfering with the intestinal barrier function and changing the intestinal microenvironment. Science of The Total Environment 2021;785:147365. 10.1016/j.scitotenv.2021.147365. 33933760

[37] Andreuzzi E, Capuano A, Poletto E, Pivetta E, Fejza A, Favero A. et al. Role of extracellular matrix in gastrointestinal cancer-associated angiogenesis. *Int J Mol Sci* 2020;21:3686–3719. 10.3390/ijms21103686. PMC7279269 32456248

[38] Kim S, Min S, Choi YS, Jo SH, Jung JH, Han K. et al. Tissue extracellular matrix hydrogels as alternatives to Matrigel for culturing gastrointestinal organoids. *Nat Commun* 2022;13:1692. 10.1038/s41467-022-29279-4. 35354790 PMC8967832

[39] Rezakhani S, Gjorevski N, Lutolf MP. Extracellular matrix requirements for gastrointestinal organoid cultures. *Biomaterials* 2021;276:121020. 10.1016/j.biomaterials.2021.121020. 34280822

[40] Zhang Y, Li X, Luo Z, Ma L, Zhu S, Wang Z. et al. ECM1 is an essential factor for the determination of M1 macrophage polarization in IBD in response to LPS stimulation. *Proc Natl Acad Sci USA* 2020;117:3083–92. 10.1073/pnas.1912774117. 31980528 PMC7022174

[41] Merchant JL . Tales from the crypts: regulatory peptides and cytokines in gastrointestinal homeostasis and disease. *J Clin Invest* 2007;117:6–12. 10.1172/JCI30974. 17200701 PMC1716224

[42] Kaymak T, Kaya B, Wuggenig P, Nuciforo S, Göldi A., Swiss EoE Cohort Study Group (SEECS) et al. IL-20 subfamily cytokines impair the oesophageal epithelial barrier by diminishing filaggrin in eosinophilic oesophagitis. *Gut* 2023;72:821–33. 10.1136/gutjnl-2022-327166. 35613844 PMC10086458

[43] Chen Y, Mai Q, Chen Z, Lin T, Cai Y, Han J. et al. Dietary palmitoleic acid reprograms gut microbiota and improves biological therapy against colitis. *Gut Microbes* 2023;15:2211501. 10.1080/19490976.2023.2211501. 37203220 PMC10202094

[44] Zhang J, Fan J, Zeng X, Nie M, Luan J, Wang Y. et al. Hedgehog signaling in gastrointestinal carcinogenesis and the gastrointestinal tumor microenvironment. *Acta Pharm Sin B* 2021;11:609–20. 10.1016/j.apsb.2020.10.022. 33777671 PMC7982428

[45] Katoh M . Genomic testing, tumor microenvironment and targeted therapy of hedgehog-related human cancers. *Clin Sci (Lond)* 2019;133:953–70. 10.1042/CS20180845. 31036756

[46] Yang M, Li J, Gu P, Fan X The application of nanoparticles in cancer immunotherapy: targeting tumor microenvironment. *Bioact Mater* 2021;6:1973–87. 10.1016/j.bioactmat.2020.12.010. 33426371 PMC7773537

[47] Barnard JA, Beauchamp RD, Russell WE, Dubois RN, Coffey RJ Epidermal growth factor-related peptides and their relevance to gastrointestinal pathophysiology. *Gastroenterology* 1995;108:564–80. 10.1016/0016-5085(95)90087-X. 7835600

[48] Xian CJ . Roles of epidermal growth factor family in the regulation of postnatal somatic growth. *Endocr Rev* 2007;28:284–96. 10.1210/er.2006-0049. 17322455

[49] Dallas NA, Fan F, Gray MJ, van Buren G II, Lim SJ, Xia L. et al. Functional significance of vascular endothelial growth factor receptors on gastrointestinal cancer cells. *Cancer Metastasis Rev* 2007;26:433–41. 10.1007/s10555-007-9070-2. 17786539

[50] Xie K, Wei D, Shi Q, Huang S Constitutive and inducible expression and regulation of vascular endothelial growth factor. *Cytokine Growth Factor Rev* 2004;15:297–324. 10.1016/j.cytogfr.2004.04.003. 15450248

[51] Almeida A, Mitchell AL, Boland M, Forster SC, Gloor GB, Tarkowska A. et al. A new genomic blueprint of the human gut microbiota. *Nature* 2019;568:499–504. 10.1038/s41586-019-0965-1. 30745586 PMC6784870

[52] Qin J, Li R, Raes J, Arumugam M, Burgdorf KS, Manichanh C. et al. A human gut microbial gene catalogue established by metagenomic sequencing. *Nature* 2010;464:59–65. 10.1038/nature08821. 20203603 PMC3779803

[53] Do TH, Nguyen TT, Nguyen TN, Le QG, Nguyen C, Kimura K. et al. Mining biomass-degrading genes through Illumina-based de novo sequencing and metagenomic analysis of free-living bacteria in the gut of the lower termite Coptotermes gestroi harvested in Vietnam. *J Biosci Bioeng* 2014;118:665–71. 10.1016/j.jbiosc.2014.05.010. 24928651

[54] Frank DN, Pace NR. Gastrointestinal microbiology enters the metagenomics era. *Curr Opin Gastroenterol* 2008;24:4–10. 10.1097/MOG.0b013e3282f2b0e8. 18043225

[55] Zundler S, Becker E, Schulze LL, Neurath MF Immune cell trafficking and retention in inflammatory bowel disease: mechanistic insights and therapeutic advances. *Gut* 2019;68:1688–700. 10.1136/gutjnl-2018-317977. 31127023

[56] Kayama H, Okumura R, Takeda K. Interaction between the microbiota, epithelia, and immune cells in the intestine. *Annu Rev Immunol* 2020;38:23–48. 10.1146/annurev-immunol-070119-115104. 32340570

[57] Lai HC, Lin TL, Chen TW, Kuo YL, Chang CJ, Wu TR. et al. Gut microbiota modulates COPD pathogenesis: role of anti-inflammatory Parabacteroides goldsteinii lipopolysaccharide. *Gut* 2022;71:309–21. 10.1136/gutjnl-2020-322599. 33687943

[58] Nowicki-Osuch K, Zhuang L, Cheung TS, Black EL, Masqué-Soler N, Devonshire G. et al. Single-cell RNA sequencing unifies developmental programs of Esophageal and gastric intestinal metaplasia. *Cancer Discov* 2023;13:1346–63. 10.1158/2159-8290.CD-22-0824. 36929873 PMC10236154

[59] Kang B, Camps J, Fan B, Jiang H, Ibrahim MM, Hu X. et al. Parallel single-cell and bulk transcriptome analyses reveal key features of the gastric tumor microenvironment. *Genome Biol* 2022;23:265. 10.1186/s13059-022-02828-2. 36550535 PMC9773611

[60] Duan ZQ, Li YX, Qiu Y, Shen Y, Wang Y, Zhang YY. et al. CD39 expression defines exhausted CD4(+) T cells associated with poor survival and immune evasion in human gastric cancer. *Clin Transl Immunology* 2024;13:e1499. 10.1002/cti2.1499. 38501063 PMC10945883

[61] Chen J, Liu K, Luo Y, Kang M, Wang J, Chen G. et al. Single-cell profiling of tumor immune microenvironment reveals immune irresponsiveness in gastric signet-ring cell carcinoma. *Gastroenterology* 2023;165:88–103. 10.1053/j.gastro.2023.03.008. 36921674

[62] Dai S, Zeng H, Liu Z, Jin K, Jiang W, Wang Z. et al. Intratumoral CXCL13(+)CD8(+)T cell infiltration determines poor clinical outcomes and immunoevasive contexture in patients with clear cell renal cell carcinoma. *J Immunother Cancer* 2021;9:e001823. 10.1136/jitc-2020-001823. 33589528 PMC7887366

[63] Chen J, Xu F, Ruan X, Sun J, Zhang Y, Zhang H. et al. Therapeutic targets for inflammatory bowel disease: proteome-wide Mendelian randomization and colocalization analyses. *EBioMedicine* 2023;89:104494. 10.1016/j.ebiom.2023.104494. 36857861 PMC9986512

[64] Chen J, Ruan X, Sun Y, Lu S, Hu S, Yuan S. et al. Multi-omic insight into the molecular networks of mitochondrial dysfunction in the pathogenesis of inflammatory bowel disease. *EBioMedicine* 2024;99:104934. 10.1016/j.ebiom.2023.104934. 38103512 PMC10765009

[65] Levi-Galibov O, Lavon H, Wassermann-Dozorets R, Pevsner-Fischer M, Mayer S, Wershof E. et al. Heat shock factor 1-dependent extracellular matrix remodeling mediates the transition from chronic intestinal inflammation to colon cancer. *Nat Commun* 2020;11:6245. 10.1038/s41467-020-20054-x. 33288768 PMC7721883

[66] Lin S, Wang J, Mukherjee PK, Mao R, West G, Czarnecki D. et al. Milk fat globule-epidermal growth factor 8 (MFGE8) prevents intestinal fibrosis. *Gut* 2024;73:1110–23. 10.1136/gutjnl-2022-328608. 38378253 PMC11248270

[67] Franzosa EA, Sirota-Madi A, Avila-Pacheco J, Fornelos N, Haiser HJ, Reinker S. et al. Gut microbiome structure and metabolic activity in inflammatory bowel disease. *Nat Microbiol* 2019;4:293–305. 10.1038/s41564-018-0306-4. 30531976 PMC6342642

[68] Halfvarson J, Brislawn CJ, Lamendella R, Vázquez-Baeza Y, Walters WA, Bramer LM. et al. Dynamics of the human gut microbiome in inflammatory bowel disease. *Nat Microbiol* 2017;2:17004. 10.1038/nmicrobiol.2017.4. 28191884 PMC5319707

[69] Li G, Lin J, Zhang C, Gao H, Lu H, Gao X. et al. Microbiota metabolite butyrate constrains neutrophil functions and ameliorates mucosal inflammation in inflammatory bowel disease. *Gut Microbes* 2021;13:1968257. 10.1080/19490976.2021.1968257. 34494943 PMC8437544

[70] Schirmer M, Denson L, Vlamakis H, Franzosa EA, Thomas S, Gotman NM. et al. Compositional and temporal changes in the gut microbiome of Pediatric ulcerative colitis patients are linked to disease course. *Cell Host Microbe* 2018;24:600–610.e4. 10.1016/j.chom.2018.09.009. 30308161 PMC6277984

[71] Hyams JS, Davis Thomas S, Gotman N, Haberman Y, Karns R, Schirmer M. et al. Clinical and biological predictors of response to standardised paediatric colitis therapy (PROTECT): a multicentre inception cohort study. *Lancet* 2019;393:1708–20. 10.1016/S0140-6736(18)32592-3. 30935734 PMC6501846

[72] Shaw KA, Bertha M, Hofmekler T, Chopra P, Vatanen T, Srivatsa A. et al. Dysbiosis, inflammation, and response to treatment: a longitudinal study of pediatric subjects with newly diagnosed inflammatory bowel disease. *Genome Med* 2016;8:75. 10.1186/s13073-016-0331-y. 27412252 PMC4944441

[73] Ma J, Wang K, Wang J, Zeng Q, Liu K, Zheng S. et al. Microbial disruptions in inflammatory bowel disease: a comparative analysis. *Int J Gen Med* 2024;Volume 17:1355–67. 10.2147/IJGM.S448359. PMC11005995 38601196

[74] Meade S, Liu Chen Kiow J, Massaro C, Kaur G, Squirell E, Bressler B. et al. Gut microbiome-associated predictors as biomarkers of response to advanced therapies in inflammatory bowel disease: a systematic review. *Gut Microbes* 2023;15:2287073. 10.1080/19490976.2023.2287073. 38044504 PMC10730146

[75] Salas A, Hernandez-Rocha C, Duijvestein M, Faubion W, McGovern D, Vermeire S. et al. JAK-STAT pathway targeting for the treatment of inflammatory bowel disease. *Nat Rev Gastroenterol Hepatol* 2020;17:323–37. 10.1038/s41575-020-0273-0. 32203403

[76] Luiz-Ferreira A, Cola M, Barbastefano V, de-Faria F, Almeida A, Farias-Silva E. et al. Healing, antioxidant and cytoprotective properties of Indigofera truxillensis in different models of gastric ulcer in rats. *Int J Mol Sci* 2012;13:14973–91. 10.3390/ijms131114973. 23203107 PMC3509623

[77] Alverdy JC, Hyoju SK, Weigerinck M, Gilbert JA The gut microbiome and the mechanism of surgical infection. *Br J Surg* 2017;104:e14–23. 10.1002/bjs.10405. 28121030 PMC7938814

[78] Shan Y, Lee M, Chang EB. The gut microbiome and inflammatory bowel diseases. *Annu Rev Med* 2022;73:455–68. 10.1146/annurev-med-042320-021020. 34555295 PMC10012812

[79] Qiu P, Ishimoto T, Fu L, Zhang J, Zhang Z, Liu Y The gut microbiota in inflammatory bowel disease. *Front Cell Infect Microbiol* 2022;12:733992. 10.3389/fcimb.2022.733992. 35273921 PMC8902753

[80] Xiong Z, Li J, Huang R, Zhou H, Xu X, Zhang S. et al. The gut microbe-derived metabolite trimethylamine-N-oxide induces aortic valve fibrosis via PERK/ATF-4 and IRE-1α/XBP-1s signaling in vitro and in vivo. *Atherosclerosis* 2024;391:117431. 10.1016/j.atherosclerosis.2023.117431. 38408412

[81] Wypych TP, Pattaroni C, Perdijk O, Yap C, Trompette A, Anderson D. et al. Microbial metabolism of L-tyrosine protects against allergic airway inflammation. *Nat Immunol* 2021;22:279–86. 10.1038/s41590-020-00856-3. 33495652

[82] Chen S, Henderson A, Petriello MC, Romano KA, Gearing M, Miao J. et al. Trimethylamine N-oxide binds and activates PERK to promote metabolic dysfunction. *Cell Metab* 2019;30:1141–1151.e5. 10.1016/j.cmet.2019.08.021. 31543404

[83] Navarro-Corcuera A, Sehrawat TS, Jalan-Sakrikar N, Gibbons HR, Pirius NE, Khanal S. et al. Long non-coding RNA ACTA2-AS1 promotes ductular reaction by interacting with the p300/ELK1 complex. *J Hepatol* 2022;76:921–33. 10.1016/j.jhep.2021.12.014. 34953958 PMC8934273

[84] Jalan-Sakrikar N, De Assuncao TM, Shi G, Aseem SO, Chi C, Shah VH. et al. Proteasomal degradation of enhancer of Zeste homologue 2 in Cholangiocytes promotes biliary fibrosis. *Hepatology* 2019;70:1674–89. 10.1002/hep.30706. 31070797 PMC6819212

[85] Aseem SO, Jalan-Sakrikar N, Chi C, Navarro-Corcuera A, de Assuncao TM, Hamdan FH. et al. Epigenomic evaluation of Cholangiocyte transforming growth factor-β Signaling identifies a selective role for histone 3 lysine 9 acetylation in biliary fibrosis. *Gastroenterology* 2021;160:889–905.e10. 10.1053/j.gastro.2020.10.008. 33058867 PMC7878301

[86] Yu FX, Meng Z, Plouffe SW, Guan KL Hippo pathway regulation of gastrointestinal tissues. *Annu Rev Physiol* 2015;77:201–27. 10.1146/annurev-physiol-021014-071733. 25293527

[87] Xie Z, Wang Y, Yang G, Han J, Zhu L, Li L. et al. The role of the hippo pathway in the pathogenesis of inflammatory bowel disease. *Cell Death Dis* 2021;12:79. 10.1038/s41419-021-03395-3. 33436549 PMC7804279

[88] An L, Cao Z, Nie P, Zhang H, Tong Z, Chen F. et al. Combinatorial targeting of hippo-STRIPAK and PARP elicits synthetic lethality in gastrointestinal cancers. *J Clin Invest* 2022;132:e155468–e155486. 10.1172/JCI155468. PMC9057599 35290241

[89] Dey A, Varelas X, Guan KL. Targeting the hippo pathway in cancer, fibrosis, wound healing and regenerative medicine. *Nat Rev Drug Discov* 2020;19:480–94. 10.1038/s41573-020-0070-z. 32555376 PMC7880238

[90] Shen H, Huang X, Zhao Y, Wu D, Xue K, Yao J. et al. The hippo pathway links adipocyte plasticity to adipose tissue fibrosis. *Nat Commun* 2022;13:6030. 10.1038/s41467-022-33800-0. 36229481 PMC9562301

[91] Guo L, Wang Y, Yang W, Wang C, Guo T’, Yang J. et al. Molecular profiling provides clinical insights into targeted and immunotherapies as well as colorectal cancer prognosis. *Gastroenterology* 2023;165:414–428.e7. 10.1053/j.gastro.2023.04.029. 37146911

[92] Zhao Y, Feng Y, Liu M, Chen L, Meng Q, Tang X. et al. Single-cell RNA sequencing analysis reveals alginate oligosaccharides preventing chemotherapy-induced mucositis. *Mucosal Immunol* 2020;13:437–48. 10.1038/s41385-019-0248-z. 31900405 PMC7181395

[93] Li L, Zhang Y, Ren Y, Cheng Z, Zhang Y, Wang X. et al. Pan-cancer single-cell analysis reveals the Core factors and pathway in specific cancer stem cells of upper gastrointestinal cancer. *Front Bioeng Biotechnol* 2022;10:849798. 10.3389/fbioe.2022.849798. 35646860 PMC9136039

[94] Stidham RW, Takenaka K. Artificial intelligence for disease assessment in inflammatory bowel disease: how will it change our practice? *Gastroenterology* 2022;162:1493–506. 10.1053/j.gastro.2021.12.238. 34995537 PMC8997186

[95] Da Rio L, Spadaccini M, Parigi TL, Gabbiadini R, Dal Buono A, Busacca A. et al. Artificial intelligence and inflammatory bowel disease: where are we going? *World J Gastroenterol* 2023;29:508–20. 10.3748/wjg.v29.i3.508. 36688019 PMC9850939

[96] Ahmad HA, East JE, Panaccione R, Travis S, Canavan JB, Usiskin K. et al. Artificial intelligence in inflammatory bowel disease endoscopy: implications for clinical trials. *J Crohns Colitis* 2023;17:1342–53. 10.1093/ecco-jcc/jjad029. 36812142 PMC10441563

[97] Chen G, Shen J. Artificial intelligence enhances studies on inflammatory bowel disease. *Front Bioeng Biotechnol* 2021;9:635764. 10.3389/fbioe.2021.635764. 34307315 PMC8297505

